# Students’ views on the block evaluation process: A descriptive analysis

**DOI:** 10.4102/curationis.v39i1.1516

**Published:** 2016-03-30

**Authors:** Ntefeleng E. Pakkies, Ntombifikile G. Mtshali

**Affiliations:** 1 School of Nursing and Public Health, University of KwaZulu-Natal, South Africa

## Abstract

**Background:**

Higher education institutions have executed policies and practices intended to determine and promote good teaching. Students’ evaluation of the teaching and learning process is seen as one measure of evaluating quality and effectiveness of instruction and courses. Policies and procedures guiding this process are discernible in universities, but it is often not the case for nursing colleges.

**Objective:**

To analyse and describe the views of nursing students on block evaluation, and how feedback obtained from this process was managed.

**Method:**

A quantitative descriptive study was conducted amongst nursing students (*n* = 177) in their second to fourth year of training from one nursing college in KwaZulu-Natal. A questionnaire was administered by the researcher and data were analysed using the Statistical Package of Social Sciences Version 19.0.

**Results:**

The response rate was 145 (81.9%). The participants perceived the aim of block evaluation as improving the quality of teaching and enhancing their experiences as students. They questioned the significance of their input as stakeholders given that they had never been consulted about the development or review of the evaluation tool, or the administration process; and they often did not receive feedback from the evaluation they participated in.

**Conclusion:**

The college management should develop a clear organisational structure with supporting policies and operational guidelines for administering the evaluation process. The administration, implementation procedures, reporting of results and follow-up mechanisms should be made transparent and communicated to all concerned. Reports and actions related to these evaluations should provide feedback into relevant courses or programmes.

## Introduction

There is world-wide awareness of the significance of investment in higher education for economic growth and social development, resulting in great emphasis on quality assurance to ensure educational relevance (World Bank 1994:1). Consequently, higher education institutions (HEIs) have executed policies and practices intended to determine whether service delivery is of good quality, as well as promoting and rewarding good teaching (Carr & Hagel 2008:153; Shah & Nair 2012:275). In South Africa, the Higher Education Quality Committee (HEQC) requires HEIs to provide proof of the mechanisms in place to monitor and evaluate teaching as part of their accreditation process, and considers students as important role players in quality promotion and assurance in higher education (Council on Higher Education [CHE] 2004:59). As such, the HEQC in South Africa, through the improving teaching and learning resource document, focuses the attention of HEIs on the importance of quality-related capacity development initiatives and internal audit systems, with student evaluation of teaching and learning (SET) as one of its endeavours.

## Background

Soliciting feedback from the students by making them evaluate instruction at class level, modules and programmes, is one of the efforts for maintaining a quality education and ensuring that their voice is more noticeable in their own education. The evaluation of learning experiences by the students, as well as the effective use of feedback originating from these evaluations could generate potential benefits to various stakeholders such as lecturers and students in the higher education context (Huxham *et al.* 2008:675; Machingambi & Wadesango 2011:169). Although several methods could be used to obtain feedback, a questionnaire according to Agbetsiafa (2010:57), appears to be used more frequently, and has been identified as the most structured, systematic, and cost-effective way of obtaining responses about instructors and courses.

Various studies have reaffirmed the main purpose of student evaluation of teaching and learning as collecting information for making two types of decisions; formative and summative (Huxham *et al.* 2008:675; Machingambi & Wadesango 2011: 167; Palmer 2012:975). Formative decisions are made by teachers to improve their teaching performance, whilst summative decisions are made by administrators to judge their personnel (Palmer 2012:976). These decisions need to be made on a fair basis because they are of crucial importance to a teacher’s career (Cleary *et al.* 2013:63). Thus, it is important to identify appropriate models or methods that best fit different educational settings and disciplines for conducting these evaluations. The primary focus of this study is SET processes for formative use.

A recent literature review by Annan *et al.* (2013:11) found that the majority of instruments specific to teaching evaluations in nursing did not seem to yield adequately reliable and valid data, were too long to complete therefore compromising the response rate, and coming short on breadth and depth as would be expected by colleges. Palmer (2012:976) noted that even well-developed, long-standing SET instruments and processes deemed fit for purpose could still require re-assessment in view of external environmental factors and/or institutional aims and objectives evolving over time, consequently posing a threat to their validity. Lekena and Bayaga (2012:274) added that flexibility should be a key feature of any lecturer or course evaluation system and that the system should provide for a range of evaluation opportunities, and allow for customisation of process and tool.

Hassanein, Abdrbo and Al Ateeq (2012:117–121) conducted a study at a nursing college in Cairo, investigating students’ evaluation as one component in the evaluation of teaching effectiveness of faculty and assessing the reliability and validity of the evaluation instrument in use. They concluded that teaching and learning was contextual by nature, and that results varied according to the diversity of teaching methods used, learning outcomes and student characteristics. As a result they recommended use of an integrated system of evaluation in the college, which would be subjected to continuous testing and refinement, that evaluations should be more dynamic, and conducted more than once a semester; for example one could be carried out a quarter into the semester, then after mid-term examination as well as two weeks before the final examination. This would allow feedback to students whilst still in that course, and also enable revisions and improvement where necessary.

A quantitative study by AbdulRaheem *et al.* (2010:15), investigating the perceived effects of students’ evaluation of teachers’ instructional practices from three Nigerian universities revealed a negative opinion about SET by junior lecturers whilst senior lecturers had a positive opinion of positive changes. The study recommended compulsory administration of SET at regular intervals in Nigerian universities.

Wen *et al.* (2011:18–23) conducted a research study at a military medical university in China to identify the effects of the evaluation of teaching on academic staff performance and the quality of the feedback. Teaching evaluation data from 2006 to 2009 were collected and analysed and students and faculty members, peer reviewers and deans were surveyed. The teaching evaluation data showed an improvement in the ratings of more than half of the faculty members in subsequent evaluations. The participants agreed that evaluations resulted in an improvement of teaching, and feedback received promptly within a week of the evaluation, was more beneficial. Faculty staff found detailed and high quality comments enlightening, providing direction on what changes to make in their teaching.

Watson (2003:148) pointed out that the feedback loop was closed when feedback provided by the students is acted upon. Harvey (2003:3) argued that in practice it was often not evident whether there was a way of closing the loop between data collection and operative action, let alone provide feedback to students on action taken. Consequently, Harvey suggested that institutions place a structure designed for identifying and delegating responsibility for action, encouraging ownership of plans of action, accountability for action taken or not taken, feedback to generators of the data and committing appropriate resources. Harvey (2003:6) perceived this as a complex task which often did not succeed, irrespective of good intentions by those who instigated the investigation.

Schiekirka *et al.* (2012: 1-2) asserted that, although empirical research on SET was in abundance, minimal focus had been directed to perceptions held by students on evaluations. As students were the ones who completed the SET questionnaires and would possibly benefit from evaluation of teaching information through improved instruction in the class room, it was important that their perceptions regarding the evaluation process be considered. Kwan (2000) stated:

[*W*]e need to understand more about how students make sense of evaluations, what influences their attitudes towards evaluations, how they react to the rating process, and what goes in their minds while making their ratings. (p. 66)

## Problem statement

The background to the study revealed the significance of SET for improving quality in education and also ensuring that the students’ voice is more noticeable in their own education. The complexity of the student feedback system, and the necessity to sustain the validity and reliability of the data collected requires the establishment of a fundamental mechanism to conduct, coordinate and monitor these surveys, as well as provide an unbiased reporting system (Palermo 2004:6). SET has been researched vastly worldwide in HEIs, especially in universities. However, there is little published work which relates to student views and their perceptions of the evaluations (Harris & Twiname 2012:146) and studies of this nature conducted in nursing education (Hassanein *et al.* 2012:118; Annan *et al.* 2013:11). Although student feedback is generally considered to be valuable, limited data addressing students’ perceptions about teaching evaluations are available from developing countries. Students in developing countries tend to be submissive, and remain unaware of their rights as stakeholders in the education process (Iqbal & Khizar 2009:69). Most importantly, according to the researcher’s knowledge, at the college where this study was conducted, students are requested to evaluate teaching at the end of every block and no research study has been conducted to elicit their views about this process since its inception.

### Research objective

The objective of the research was to analyse and describe the views of students on block evaluation and how feedback from the process is managed.

### Contribution to the field

The findings of this study highlighted the gaps in the block evaluation process and, therefore, an increasing operational understanding which could be applied in future by nursing colleges and similar establishments. Lecturers at the college of the study should reflect on its findings and recommendations, thereby enhancing their personal development. In addition, the study should serve as a baseline for future research studies for nursing colleges and relevant higher education institutions.

## Definition of key concepts

### Block evaluation

This is a process that occurs at the end of a teaching block. Students are requested to complete a standard questionnaire eliciting their views about teaching and learning activities of that block with the aim of improving its quality. The block comprises of lectures, tutorials, practical demonstrations and written assessments.

### Feedback

Feedback refers to expressed opinions of students about their lecturer’s teaching and learning collected at end of block.

### Feedback process

The process by which students and lecturers are informed of the results of the evaluation.

## Research method and design

### Design

A quantitative, non-experimental, descriptive research design was adopted for this study to analyse and describe the views of nursing students on block evaluation, and how feedback obtained from this process was managed. This was part of a larger study that elicited perceptions from lecturers and students regarding the evaluation process. The conceptual framework that guided the study was Harvey’s (2003:4) model of a full feedback-action cycle, that is ‘The Satisfaction Cycle’. The whole study utilised all the concepts of the model; however, the ones applicable for this report are stakeholder determined questions, questionnaire distribution, implementation and monitoring, and feedback to stakeholders.

### Study Context

KwaZulu-Natal, where the study was conducted, is one of nine provinces in South Africa. Nursing education is provided by universities as well as nursing colleges. The nursing college for the study falls under the provincial Department of Health and was affiliated to two universities at the time of the study. Students’ evaluation of teaching, known as block evaluation in that institution, was introduced approximately 15 years ago to provide lecturers with feedback in order to improve the quality of teaching and student learning. At the end of the block, students are requested to complete a standard questionnaire eliciting views about the quality of teaching and learning activities of that block. To the researcher’s knowledge, no scientific enquiry had ever been carried out regarding block evaluation of teaching since its inception.

### Population and sampling

The population for the study consisted of 177 students, 83 of whom were registered for the four-year Diploma in Nursing (General, Psychiatric, Community) and Midwifery (R425), whilst 94 were registered for the two-year bridging course leading to registration as a General Nurse (R683). Inclusion criterion for this study was participation in at least three students’ evaluations of the teaching and learning process. This was to ensure that the students had adequate exposure and observation of the process at different levels of their training. No sampling was performed and the entire population was used. This allowed the researcher to obtain data from all readily available individuals for the study and also benefit from the diversity provided by the cohort.

### Data collection instrument

Data was collected through a self-administered questionnaire. The questionnaire was developed by the researcher guided by the conceptual framework, research objectives, studies by Colford (2007) and Chang (2001), as well as relevant literature. The instrument had two sections.

Section A of the instrument covered demographic questions. Section B had five subsections which consisted of purpose of the evaluation, development and review of the evaluation tool, components, administration of the tool, feedback and its management. The four subsections of section B consisted of questions with responses to select from, as well as space for additional information. In the last subsection, participants indicated their responses on a Likert scale of 1 to 5 where: 1 = strongly disagree, 2 = disagree, 3 = neither agree nor disagree, 4 = agree and 5 = strongly agree. The last question was an open-ended question which requested students for any additional suggestions on how the process could be improved.

### Validity and reliability

The validity of the instrument used in this study was determined through content validity. The researcher checked the items in the data collection instrument against the conceptual framework and research objective to determine whether they measured all of the elements of interest in the study. In addition, the questionnaire was subjected to scrutiny by a group of experts in nursing education and research at the university as well as a statistician. Minor adjustments to the tool were made according to their suggestions.

The reliability of the instrument was ascertained by a test-retest method administered at an interval of two weeks amongst four students who were in the first year level and did not fit the inclusion criteria, and were not included in the main study. The consistency of the responses between the two tests was scrutinised, and the researcher obtained the same results from the two administrations of the instrument confirming reliability.

#### Data collection method

After obtaining ethical approval and permissions, the principal of the college was approached in order to gain access to the students who were eligible for the study. With permission and assistance from the responsible lecturers, a day was arranged where the researcher met with all the potential participants and explained the nature and purpose of the study, and the rights of participants. The researcher then arranged days for data collection according to student availability. Some students were on block, whilst, for some, arrangements were made for data collection to coincide with their day off at the college. Written consent for participation in the study was sought before the questionnaire was handed to the participants. This was carried out during their lunch time away from their classrooms with the assistance of class representatives as the researcher was also a lecturer in the campus and known by the students. The participants were requested to complete and drop the questionnaires into a box which was placed in their rest room. The researcher emptied the box at the end of each day. The data collection process took approximately two weeks in May 2012.

### Data analysis

Data were analysed using the Statistical Package for Social Science (SSPS), version 19, with the assistance of a statistician. Descriptive statistics were employed with the use of frequency, percentage distributions, mode, median and standard deviations. Data from open-ended questions were used to support results from the close-ended questions.

## Ethical consideration

Prior to conducting the study, the research proposal was presented to the University of KwaZulu-Natal Ethics Committee and approval was received (HSS/1243/011M). Permission was also obtained from the KwaZulu-Natal College of Nursing, the KwaZulu-Natal Department of Health (HRM 008/11) and the principal of the selected nursing college.

To obtain the participant’s approval, a letter explaining the purpose and nature of the study was given to each participant and written informed consent was obtained. The participants were informed that they could withdraw from the study at any time if they no longer wanted to participate. They were informed that they would not receive any monetary benefits for completing the questionnaires. All participants were assured that no information given by them would be shared with other persons without their authorisation, in order to ensure confidentiality. Furthermore, participants’ identities were protected as no names were written on questionnaires that could identify respondents; code numbers were instead used to ensure anonymity.

## Results

Questionnaires were administered to 177 students and 145 were returned completed. The response rate was therefore 81.9%.

### Section A: Demographic profile

Table 1 shows that the majority of the participants 116 (80%) were females. All the participants (145) indicated their age, the majority of whom, 86 (59.3%), were between 20 and 29 years of age. The minimum age was 20 years, with the median of 29 and the maximum 59. The majority of students, 104 (71.7%), were at the second year level of their training, which included a four year diploma course and two year bridging course students.

**TABLE 1:** Demographic profile (*n* = 145).

Variable	*F*	Percentage
**Gender**		
Male	29	20
Female	116	80
**Age (in years)**		
20–29	86	59.3
30–39	44	30.3
40–49	13	9
50–59	2	1.4
**Course of study**		
Diploma	69	47.6
Bridging	76	52.4
**Year of Study**		
2	104	71.7
3	10	6.9
4	31	21.4

*Source*: Pakkies, E.N. & Mtshali, N.G., 2013, ‘Exploring students’ evaluation of the teaching and learning process at a selected nursing campus in Kwazulu-Natal: Lecturers’ and students’ perspective’, unpublished Master’s thesis, School of Nursing and Public Health, University of KwaZulu-Natal

*F*, frequency; *R*, ranking according to popularity; SD, standard deviation.

### Section B: The purpose of the evaluation of teaching

The participants were provided with six items to select what they perceived as the purpose of the evaluation and were allowed to make multiple selections. The majority, 128 (83.3%), selected, ‘To improve the quality of teaching’, closely followed by, ‘To improve student’s learning experience’, 117 (80.7%). According to 88 (60.7%), the institution conducted evaluations of teaching ‘To satisfy institutional requirements’. To support this assertion, 29 (20.0%) stated that most lecturers do not give feedback following an evaluation, which could mean that they did not care; in effect, one student commented, ‘I doubt if anyone even cares to read our comments’. Table 2 illustrates the rest of the selections, indicating the item ranking according to popularity.

**TABLE 2:** The purpose of the evaluation of teaching.

Purpose	*F*	%	*R*	Mean	SD
To improve the quality of teaching	128	88.3	1	1.12	0.323
To identify staff development needs	96	66.2	3	1.34	0.475
To improve student’s learning experience	117	80.7	2	1.19	0.396
To provide opportunity for democratic practices	88	60.7	4	1.39	0.49
To increase student learning motivation	86	59.3	5	1.41	0.493
To satisfy institutional requirements	88	60.7	4	1.39	0.49

*Source*: Pakkies, E.N. & Mtshali, N.G., 2013, ‘Exploring students’ evaluation of the teaching and learning process at a selected nursing campus in Kwazulu-Natal: Lecturers’ and students’ perspective’, unpublished Master’s thesis, School of Nursing and Public Health, University of KwaZulu-Natal

*F*, frequency; *R*, ranking according to popularity; SD, standard deviation.

### Development or review of the evaluation tool

None of the respondents reported having been involved in the development or review of the evaluation tool. When asked if they thought it was necessary to be involved, the majority, 140 (96.6%), saw it as necessary, whilst 53 (36.6%) affirmed that this would have enabled them to contribute towards the contents of the questionnaire. Forty-eight (33.1%) indicated that their participation would have enabled them to contribute towards other aspects of the administration process. A further 38 (26.2%) of the respondents considered the current tool inadequate for addressing their concerns, but failed to elaborate on the perceived inadequacy. Five respondents did not indicate a need to be involved and did not give reasons for their choice.

### Components of the evaluation

The participants were then asked to select what they considered as components of the existing evaluation tool from listed items. They were allowed to select multiple items and were also provided with space to add more items if necessary. As indicated in table 3, the following items were the top two; ‘Appropriateness of teaching methods’ 136 (93.8%), followed by ‘Feedback following assessment’ 116 (80%), and the rest as indicated in the table. They were further requested to indicate items they perceived as important and preferred to be included. It is interesting to note that, ‘Support and guidance provided’ featured strongly as favourites by the participants, 119 (82.6%) followed by, ‘Lecturer’s teaching skills, 112 (77.8%). As depicted in the last three columns of table 3, ‘Appropriateness of teaching methods‘ and ‘Lecturer’s teaching skills’ ranked within the top three positions for both actual and preferred items for the participants. Similarly, a lecturer’s personal characteristics was the least popular for both actual and preferred. It was interesting to note that although 53 (36.6%) of the participants expressed a desire to contribute on what the contents of the questionnaire should be, no suggestions were made although space was provided.

**TABLE 3:** Components of the evaluation tool.

Components	Actual			Preferred		
	
	*F*	%	*R*	*F*	%	*R*
1. Lecturer’s personal characteristics	15	10.3	**10**	57	39.3	**10**
2. Lecturer’s teaching skills	114	78.6	**3**	112	77.8	**2**
3. Appropriateness of teaching methods	136	93.8	**1**	107	74.3	**3**
4. Support and guidance provided	100	69	**5**	119	82.6	**1**
5. Relationship between students and lecturer	49	33.8	**8**	77	53.5	**8**
6. Assessment of learning	106	73.1	**4**	99	68.8	**5**
7. Feedback following assessment	116	80	**2**	106	73.6	**4**
8. The overall rating of the lecturer	24	16.6	**9**	84	58.3	**7**
9. Workload (subject content)	77	53.1	**6**	106	73.6	**4**
10. Course material	64	44.1	**7**	76	53.1	**9**
11. Students’ self-evaluation of learning	64	44.1	**7**	89	61.8	**6**

*Source*: Pakkies, E.N. & Mtshali, N.G., 2013, ‘Exploring students’ evaluation of the teaching and learning process at a selected nursing campus in Kwazulu-Natal: Lecturers’ and students’ perspective’, unpublished Master’s thesis, School of Nursing and Public Health, University of KwaZulu-Natal

*F*, frequency; *R*, ranking according to popularity.

### Administration of the evaluation tool

The majority of participants, 131 (90.3%), reported that the evaluation takes place at the end of the block, and 142 (97.9) indicated that the class teacher distributes the evaluation tool. Responding to when they thought was the best time to evaluate teaching, 63 (43%) preferred the end of block, whilst 42 (29%) felt that it should be carried out both in the middle of the block and at the end of the module, with a few suggesting that the latter would allow changes to be made where necessary. The rest of the participants’ choices were between the middle and the end of module.

Just above half of the students 74 (51%) were of the view that the distribution and collection of the evaluation tool should be the responsibility of the class teacher, whilst 29 (20%) thought this task should be that of the subject teacher. Twenty seven students (18.6%) considered the head of department an ideal person, whilst 12 (8.3%) suggested that a non-teaching college staff member was more appropriate. Only two participants opted for a student representative for the task. One participant did not answer this question. Fifteen (10.3%) commented that they often had a fear of repercussions that could arise if a lecturer (who received a negative evaluation) could identify their handwriting, especially in small classes.

## Feedback

The participants were asked if they received feedback following the evaluation they participated in. Different opinions were revealed on this issue as shown in table 4. Fifty-three (36.6%) reported receiving feedback whilst 51 (35.4%) stated that they sometimes received feedback. However, 40 (27.8%) asserted that feedback was not given following a block evaluation.

**TABLE 4:** Do students receive feedback?

Do student receive feedback?	*F*	%
Yes	53	36.6
No	40	27.8
Sometimes	51	35.4

*Source*: Pakkies, E.N. & Mtshali, N.G., 2013, ‘Exploring students’ evaluation of the teaching and learning process at a selected nursing campus in Kwazulu-Natal: Lecturers’ and students’ perspective’, unpublished Master’s thesis, School of Nursing and Public Health, University of KwaZulu-Natal

*F*, frequency.

**TABLE 5:** When and how is feedback communicated to students?

Variable	Actual practice		Preferred	
	
	*F*	%	*F*	%
**When do you receive feedback**				
On the last day of that block	49	33.8	64	44.1
Within 2 weeks of the evaluation	10	6.9	38	26.2
Beginning of the following block	40	27.3	34	23.4
Other	44	30.3	7	4.8
Missing	2	1.4	2	1.4
**Who gives you the feedback**				
Class teacher	104	71.7	60	41.4
Subject teachers	1	1.7	48	33.1
Subject head (HOD)	-	-	20	13.8
Student representatives	-	-	2	1.4
Other	40	27.6	15	10.3
**How is feedback given**				
Verbally	107	73.8	107	73.8
Written	-	-	21	14.5
Other	38	26.2	9	6.2
Missing	-	-	8	5.5
Other	38	26.2	9	6.2
Missing	-	-	8	5.5

*Source*: Pakkies, E.N. & Mtshali, N.G., 2013, ‘Exploring students’ evaluation of the teaching and learning process at a selected nursing campus in Kwazulu-Natal: Lecturers’ and students’ perspective’, unpublished Master’s thesis, School of Nursing and Public Health, University of KwaZulu-Natal

*F*, frequency.

The majority of students, 107 (73.8%), indicated that feedback was communicated verbally to them, whilst 104 (71.7%) indicated that it was done by the class teacher. Forty four respondents (30.3%) selected the option ‘other’, further explaining in the open section that they have never received any feedback. A further 49 (34.3%) reported receiving feedback on the last day of the block, whilst 40 (28%) revealed that they received feedback at the beginning of the following block. A few participants 10 (6.9%), indicated having received feedback within two weeks of the evaluation. The majority of the participants 60 (41.4%) indicated that they preferred feedback to be communicated by their class teachers, whilst 48 (33.1%) preferred the subject teacher. Two participants selected a student representative for that task. Regarding the mode of communication, the majority, 107 (73.8%), had no objection to feedback being communicated verbally whilst 21 (14.5%) preferred a written report.

The majority of the students, 64 (44.8%), were of the opinion that the last day of the block would be more suitable to receive feedback, whilst 38 (26.6%) preferred feedback within two weeks of the evaluation. Thirty-four participants (23.8%) considered the beginning of the following block acceptable. Seven students (4.9%) indicated no preference with regard to when they should receive feedback.

### Are there any changes that have occurred as a result of the evaluation?

In response to this question, the majority of students, 82 (57.3%), reported that no changes had occurred as a result of the evaluation. Nevertheless, 61 (42.7%) reported that changes had occurred. From the participants who identified changes, 21 (14.5%) described them as ‘minor’. Some of the students reported an improvement of teaching strategies in the classroom supplementation of lecture method with role plays and case studies by some lecturers. Some provided more guidance to students with projects, more time was allocated for practical skills, research and group discussion time in-between lectures. However, most students did not elaborate on the nature of changes made.

## Discussion

Steyn (2011:79) identifies knowledge and understanding of the purpose of students’ evaluation of teaching as essential in order to enhance participant enthusiasm as well as guide its implementation. For stakeholders to make a significant contribution to the evaluation of the teaching and learning process, it is necessary that its purpose is as precise and distinctive as possible.

The current study revealed that the majority of students believed that their college asked them to evaluate their lecturers in order to improve the quality of teaching (88.3%), and to improve students’ learning experience (80.7%). These results resonate with findings from previous studies. For example, Schiekirka *et al.* (2012:4) reported that the medical students from their qualitative study perceived the evaluation of teaching as an important tool for improving the teaching processes, and outcomes. These respondents believed that participation in the evaluations not only allowed them to express their opinion about their courses, but also provided lecturers with ways of assessing whether learning objectives had been met in a specific course.

The present study also revealed that students were not involved in the development or review of the evaluation of a teaching tool. This is consistent with Harris and Twiname’s (2012:146) contention that students are amongst the main actors extremely engaged in most institutions’ assessment process, yet they are constantly disregarded from such consultations and examinations. Almost all the students, (97.2%) were of the opinion that they should be involved in the designing or reviewing of the block evaluation tool, which included deciding on items that should be in the questionnaire. In support of student inclusion in the development and review process, Catano and Harvey (2011:702) pointed out that SET instruments are frequently developed from specialist academic staff opinion, and, despite the fact that they could be suitable and illuminating, may fall short of portraying vital aspects of teaching and courses from a student’s point of view. It was also noted that students were more likely to provide well thought-through information about issues perceived as important by them as opposed to those defined by the institutions (Shah & Nair: 2012:277).

It was also suggested by 28.6% of the participants that the evaluation takes place in the middle and at the end of the block so that problems or concerns, if any, are discovered early whilst it was still possible to institute changes. These findings were similar to those reported by Colford (2007:2–3), who reported a majority of the students in a modular system stating that it would allow implementation of improvements for the remainder of that module. The students who participated in the study conducted by Harris and Twiname (2012:155) also identified the timing of evaluations as problematic, as evaluations carried out at the end of the semester would only be of benefit to future students and not to those who participated in the evaluation.

Following evaluations amongst the participants, findings of this study revealed disparate views regarding feedback. Although some of them reported having received feedback, the process appeared conspicuously inconsistent. In line with these findings, Colford’s (2007:2) participants reported lack of feedback regarding their evaluations of teaching. Watson (2003) cautioned that if students became accustomed to completing evaluation questionnaires, but do not receive feedback from the process, they could be less inspired to take part in future evaluative processes. Likewise, Spiller and Harris (2013:258) expressed concern that students could become ‘cynical and disaffected’ should they feel that their voices were not being heard, and equally weary that the evaluation process could end up as a meaningless ritual without any result with little or no impact on student learning experiences. About (60, 7%) of the students in the current study reported that the institution was eliciting their views on the evaluation process just ‘to satisfy institutional requirements’. This could be an indication of an element of *doubt* from students that lecturers and the institution take the process seriously, hence there was no feedback.

More than half of the students (57.3%), reported not being aware of any changes that occurred as a result of their evaluation, whilst some changes were reported by 42.7% of the students. In contrast, university teachers from Moore and Koul’s (2005:66) study admitted making changes to their teaching and subjects as a result of student feedback but the extent to which changes were made varied. Likewise, in Van Wyk and McLean’s (2007:28) study, participants pointed out that qualitative comments from students’ feedback in particular provided specific direction for improvement on their shortcomings as facilitators. Nonetheless, it could be postulated that from the current study there could still be a possibility that changes do occur as a result of student’s input but would have an impact only on the next group of students.

In a study by Al-Kuwaiti (2015:170), both instructors and students agreed that participation in faculty development workshops would probably improve teaching skills. Spiller and Harris (2013:266–267), citing previous literature, suggested that difficulties associated with interpreting regular survey data contributed to the failure in proper utilisation of feedback from students by instructors. In their enquiry into academic staff engagement and use of student feedback, Spiller and Harris concluded that although some of the participants in their study reported problems with interpretation of data, they did not seek assistance to enable utilisation of the results. Recommendations from that study included provision of resources and support for proper data analysis, interpretation and implementation process for academics.

## Limitations of the study

The current study was confined to students at a specific nursing campus where the evaluation process might be different from other institutions, and therefore findings cannot be generalised to other entities. The researcher was a lecturer at the campus at the time of the study, which might have influenced the students’ responses.

## Conclusion

According to Meng, Chea, and Nooi (2012:2), a successful evaluation process utilises a questionnaire with items that are relevant for purpose and agreed upon by the student evaluators and the lecturers to be evaluated. The findings of this study revealed that students were not involved in the development and evaluation of the block evaluation tool and felt that it was important to make a contribution on items that should be in the evaluation tool. This indicates their enthusiasm to be incorporated as active partners in the process in order to improve teaching and learning. Most students did not believe that the process was taken seriously by their lecturers and the institution because they rarely received feedback on evaluations they participated in. It also emanated from the study that changes as a result of the block evaluations were minimal, if they ever occurred. Furthermore, participants doubted the existence of guidelines to ensure the proper management of data obtained from the evaluations.

## Recommendations

Taking the results and conclusion into consideration, the authors recommend that the college management addresses the identified gaps by establishing a clear organisational structure with supporting policies and operational guidelines for administering the evaluation process. In line with a suggestion by Spiller and Harris (2013:267), lecturers should be provided with more guidance on how to respond to the evaluations given by their students as they might not know how to act on the feedback. The administration, implementation procedures, reporting of results and follow-up mechanisms should be transparent and communicated to all concerned. These evaluations should feed back into relevant course or programme evaluation committees.

## References

[CIT0001] AbdulRaheem Y., AbdulRahman A.U., Ayorinde A.S. & Olubode O.C, 2010, ‘University teachers’ perception of the effects of students evaluation of teaching on lecturers instructional practices in Nigeria’, paper presented at the first international conference of collaboration of education faculties in West Africa (CEFWA), Ilorin, 9–11 February.

[CIT0002] Agbetsiafa D, 2010, ‘Evaluating effective teaching in college level economics using student ratings of instruction: A factor analytic approach’, *Journal of College Teaching & Learning* 7(5), 57–66.

[CIT0003] Al-Kuwaiti A, 2015, ‘Students evaluating teaching effectiveness process in Saudi Arabian medical colleges: A comparative study of students’ and faculty members perception’, *Saudi Journal of Medicine & Medical Sciences* 2(3), 166–172.

[CIT0004] Annan S.L., Tratnack S., Rubenstein C., Metzler-Sawin E. & Hulton L, 2013, ‘An integrative review of student evaluations of teaching: Implications for evaluation of nursing faculty’, *Journal of Professional Nursing* 29(5), e10–24. 10.1016/j.profnurs.2013.06.004 24075267

[CIT0005] Carr R. & Hagel P, 2008, ‘Students evaluation of teaching quality and their unit online activity: An imperical investigation’, viewed 17 July 2011, from http://ascillate.org.au/conferences/melbourne08/carr-r.pdf

[CIT0006] Catano V.M. & Harvey S, 2011, ‘Student perception of teaching effectiveness: Development and validation of the evaluation of teaching competencies scale’, *Assessment & Evaluation in Higher Education* 36(6), 701–717. 10.1080/02602938.2010.484879

[CIT0007] Chang T.S, 2001, ‘Teachers college faculty attitudes toward student ratings: The comparison between required policy and optional policy’, *American Psychological Association (APA)*, annual meeting of APA, San Francisco, 24-28th August.

[CIT0008] Council on Higher Education (CHE), 2004, Improving teaching and learning resource, CHE, Pretoria.

[CIT0009] Cleary M., Happell B., Lau S. & Mackey S, 2013, ‘Student feedback on teaching: Some issues for consideration for nurse educators’, *International Journal of Nursing Practice* 19 (1), 62–66. 23425381 10.1111/ijn.12018

[CIT0010] Colford J, 2007, ‘Student evaluation of modules: A student perspective’, research report, *Quality in Business Education*, UK.

[CIT0011] Harris T. & Twiname L, 2012, ‘Student evaluations of teaching: The students’ perspective’, *Studies in Learning, Evaluation Innovation and Development* 9(1), 145–156.

[CIT0012] Harvey L, 2003, ‘Student feedback’, *Quality in Higher Education* 9 (1), 3–20.

[CIT0013] Hassanein S., Abdrbo A. & Al Ateeq E, 2012, ‘Student evaluation of faculty at college of nursing’, *International conference on management and education innovation, International proceedings of economics development and research (IPEDR)*, 37, IACSIT Press, Singapore http://www.ipedr.com/list-6-1.html

[CIT0014] Huxham M., Laybourn B., Cairncross S., Gray M., Brown N., Goldfinch J. et al., 2008, ‘Collecting student feedback: a comparison of questionnaire and other methods’, *Assessment & Evaluation in Higher Education* 33(6), 675–686.

[CIT0015] Iqbal M. & Khizar B, 2009, ‘Medical students’ perceptions of teaching evaluations’, *The Clinical Teacher* 6(2), 69–72.

[CIT0016] Kwan K.P, 2000, ‘How university students rate their teachers: A study of the rating attitudes and behaviours of university students in teaching evaluations’, unpublished PhD thesis, University of Durham.

[CIT0017] Lekena L.L. & Bayaga A, 2012, ‘Quality assurance in education: Student evaluation of teaching’, *International Journal of Educational Sciences* 4(3), 271–274.

[CIT0018] Machingambi S. & Wadesango N, 2011, ‘University lecturers’ perceptions of students evaluation of their instructional practices’, *Anthropologist* 13(3), 167–174.

[CIT0019] Meng L.T., Chea C.C. & Nooi P.S 2012, ‘Questionnaire design and data analysis: An alternative approach in student evaluation of teaching (SET)’, *Sunway Academic *Journal**, 9.

[CIT0020] Moore S. & Kuol N, 2005, ‘Students evaluating teachers: Exploring the importance of faculty reaction to feedback on teaching’, *Teaching in Higher Education* 10(1), 57–73.

[CIT0021] Palermo J, 2004, *Closing the loop on student evaluations. ATN quality improvement group*, viewed 29 March 2011, from http://www.aair.org.au/jir/2004Papers

[CIT0022] Palmer S, 2012, ‘The performance of a student evaluation of teaching system’, *Assessment & Evaluation in Higher Education* 37(8), 975–985.

[CIT0023] Pakkies E.N. & Mtshali N.G, 2013, ‘Exploring students’ evaluation of the teaching and learning process at a selected nursing campus in Kwazulu-Natal: Lecturers’ and students’ perspective’, unpublished Master’s thesis, School of Nursing and Public Health, University of KwaZulu-Natal.

[CIT0024] Shah S.N. & Nair M, 2012, ‘The changing nature of teaching and unit evaluations in Australian uuniversities’, *Quality Assurance in Education* 20(3), 274–288.

[CIT0025] Schiekirka S., Reinhardt D., Heim S., Fabry G., Pukrop T., Anders S. et al, 2012, ‘Student perceptions of evaluation in undergraduate medical education: A qualitative study from one medical school’, *BMC Medical Education* 12(45), 1–7. 22726271 10.1186/1472-6920-12-45 PMC3408338

[CIT0026] Spiller D. & Harris T, 2013, ‘Learning from evaluations: Probing the reality’, *Issues in Educational Research, Special Issue* 23(2), 258–268

[CIT0027] Steyn J, 2011, ‘An assessment of the process of student evaluation of teaching effectiveness’, mini-dissertation, MPhil, Faculty of Management, University of Johannesburg.

[CIT0028] Van Wyk J. & Mclean M, 2007, ‘Maximizing the value of feedback for individual facilitator and faculty development in a problem-based learning curriculum’, *Medical Teacher* 29(1), e26–31. 17538828 10.1080/01421590601032435

[CIT0029] Watson S, 2003, ‘Closing the feedback loop: Ensuring effective action from student feedback’, *Tertiary Education and Management* 9,145–157.

[CIT0030] Wen S., Xu J., Carline J., Zhong Y. & Shen S, 2011, ‘Effects of a teaching evaluation system: A case study’, *International Journal of Medical Education* 2, 18–23.

[CIT0031] World Bank, 1994, ‘Higher education: The lessons of experience’, *Development in Practice Series*, World Bank, Washington, D.C.

